# Sports Diet and Oral Health in Athletes: A Comprehensive Review

**DOI:** 10.3390/medicina60020319

**Published:** 2024-02-13

**Authors:** Antina Schulze, Martin Busse

**Affiliations:** General Outpatient Clinic of Sports Medicine, University of Leipzig, 04103 Leipzig, Germany; drmartinbusse@gmail.com

**Keywords:** sports diet, athletes, oral health, caries, periodontitis, nutrition, dental health

## Abstract

Food and fluid supply is fundamental for optimal athletic performance but can also be a risk factor for caries, dental erosion, and periodontal diseases, which in turn can impair athletic performance. Many studies have reported a high prevalence of oral diseases in elite athletes, notably dental caries 20–84%, dental erosion 42–59%, gingivitis 58–77%, and periodontal disease 15–41%, caused by frequent consumption of sugars/carbohydrates, polyunsaturated fats, or deficient protein intake. There are three possible major reasons for poor oral health in athletes which are addressed in this review: oxidative stress, sports diet, and oral hygiene. This update particularly summarizes potential sports nutritional effects on athletes’ dental health. Overall, sports diet appropriately applied to deliver benefits for performance associated with oral hygiene requirements is necessary to ensure athletes’ health. The overall aim is to help athletes, dentists, and nutritionists understand the tangled connections between sports diet, oral health, and oral healthcare to develop mitigation strategies to reduce the risk of dental diseases due to nutrition.

## 1. Introduction

The athlete should be aware of the risks associated with a sports-specific diet, especially concerning the health status of the oral cavity. Alterations and oral diseases negatively impact general health, well-being, and physical performance [1]. Nutrition is of major importance in managing and enhancing athletic performance and post-workout nutritional recommendations are fundamental for recovery and adaptive processes [2,3,4]. Low-energy intakes can result in a loss of muscle mass, weak bone structure, hormonal dysfunction, performance drop, and increased risk of injuries [5]. During times of high physical activity, carbohydrate and protein intake is particularly important to control body weight and to maximize training effects, glycogen storage, muscle gain, and tissue repair [5]. At the same time, sports nutrition can have a major impact on oral health due to increased consumption of sports drinks, energy bars, and gels [6,7]. Increased intake, frequent consumption moments with dental exposure to carbohydrate-rich foods, free sugars, sports nutrition products, and acidic and carbohydrate-containing sports drinks increase the risk of dental erosion, dental caries, and inflammatory periodontal disease, especially in cases of dehydration and poor oral hygiene [8]. A contemporary lifestyle with suboptimal nutritional quantity and quality contribute to an increased risk of tooth decay, meanwhile synthetically produced foods are of particular concern [9]. Untreated caries and deep cavities can trigger a disseminated infection, affecting the well-being and performance of athletes [10,11]. Dental pain has been described as the cause of up to 18% of loss of performance [11]. In particular, elite athletes with a weakened immune system and high cortisol levels due to stress, demand, and overload, in combination with additional dental and/or periodontal inflammation, are at risk [11,12]. Teeth can be seen as the window to overall systemic health.

Our goal is to present a comprehensive overview and to offer practical recommendations for clinicians and for athletes to improve their dental awareness and knowledge about nutritional risks for oral health. 

## 2. Prevalence of Dental Diseases in Athletes: Consequences and Reasons

### 2.1. Dental Caries in Athletes

The data search for this comprehensive review concerning athletes’ oral health was limited to elite or professional athletes. Outcome measures of oral health concerning the prevalence, incidence, and/or severity of dental caries, dental erosion, gingivitis, and periodontal disease were included. Only studies with clinical and/or radiological data were considered. The final included 17 studies represented a wide range of sports and were mostly from Europe (65%). Caries was evaluated in 15 studies (88%) and recorded as DMF(T) (decayed, missing, and filled teeth) and/or as the proportion of athletes with caries and treatment provided in dental clinics at larger events (Table 1). The lack of consistency in the outcome measures between the studies made the comparison of results difficult. Many studies did not conduct an examiner calibration or training for data validity. Most of the studies were epidemiological surveys, where caries incidence ranged from 15% to 89% of evaluated athletes, whereas the worldwide estimated prevalence of caries in athletes is reported as 46% [13]. The National Institute of Dental and Craniofacial Research Health reported untreated dental caries in 29.3% of adults and a DMFT of 6.7 (ages 20–34 years) [14]. DMF-T values of elite athletes (ages 21–27 years) in industrialized countries ranged from 2.7 to 5.7 [15,16,17,18,19]. Worse conditions were found among German triathletes (DMFT = 9.7; mean age: 37 years) [20] and soccer players from Thailand (DMFT = 10.1; mean age: 27.5 years) [21], with respect to a high age-dependence of this parameter, and in Olympic athletes with Down syndrome (DMFT = 10.2) [22] (Table 1).

Reports from the London 2012 Olympic Games revealed poor oral health, including caries (55%) in 278 athletes from various countries who visited a dental clinic [10]. The study results from the Rio 2016 Olympic Games showed that 50% of Dutch athletes needed dental treatment and stated that regular dental check-ups are necessary to ensure that the athletes are healthy during competitions [18]. In 2018, a sample of athletes from the United Kingdom from different sports areas was examined and 49% presented established caries [27]. Overall, the lack of radiographic examination can result in an underestimated prevalence. One study used only X-ray interpretations to measure the caries prevalence in athletes from the Middle East [28]. A recent study in Russia showed a lower quality of previous caries treatments via X-rays and more frequent self-reported gingival and periodontal diseases in 132 Olympic athletes compared to 104 residents of similar age [31]. The lack of comparative groups within studies is another common use. Only three studies [15,20,26] compared their results with non-athletes and reported a similar caries prevalence, which is contrary to other studies using no representative matched control groups/populations [10,25]. One case–control study reported a significantly higher caries experience (DMFT 5.54 vs. 2.14) in rugby players [18]. Among athletes, a significant correlation between training volume and caries prevalence was found [20]. Two studies with normal athlete controls showed that caries levels in swimmers and controls were similarly low [26] and higher in endurance sports (34% vs. 19%) [17]. Three studies compared data with population data [15,16,19]. However, without an age-matched control group, it is difficult to assess the oral health risk in elite sports. Disease incidence was generally not clearly differentiated by the socioeconomic status but appears to affect athletes from developing and developed countries.

### 2.2. Dental Erosion in Athletes

The prevalence of dental erosion, measured as BEWE (basic erosive wear examination) or as ETW (erosive tooth wear), was in the range of 36–85%, as reported in six studies. Two studies compared the data with a population survey. The prevalence of dental erosion in Dutch athletes before the Olympic Games was 59% and twice as high as Dutch non-athletes of a similar age [16]. A second study found a prevalence of 42% in athletes vs. 52% in a population [32] including disadvantaged people. The mean prevalence of erosion in athletes has been estimated as 47% [33]. Compared to non-exercising controls, a significantly higher risk of dental erosions was reported in triathletes [20], but not for competitive athletes in a sample of predominantly runners, biathlon athletes, and skiing athletes [17]. In contrast to caries assessment, the use of BEWE measures might be biased by diagnostic uncertainties but is the most recommended scoring system for dental erosions [34].

### 2.3. Periodontal Disease (Gingivitis and Periodontitis) in Athletes

Gingivitis occurs due to plaque accumulation along the gingival margins. As it progresses, there can be an inflammatory destruction of the periodontal ligament (periodontitis).

Gingivitis was evaluated in nine studies based on gingival index (GI), bleeding on probing (BOP), and periodontal bleeding index (BPI).

Periodontitis was measured in nine studies based on basic periodontal examination (BPE), periodontal pocket depth (PPD), and periodontal screening index (PSI). 

Gingivitis prevalence among elite athletes was between 58% and 77% [10,16,18,19,27]. Only in one study, using rugby players, was gingival health compared to non-exercising controls [18], with significantly worse gingival findings in athletes.

During the London 2012 Olympic Games, the prevalence of moderate to severe periodontal disease was up to 15% [10]. Data from controls or population norms were mostly not presented. A comparison between competitive and non-competitive endurance athletes showed a higher prevalence of periodontitis (40% vs. 12%) in the competitive athletes [17]. Gallagher et al. [27] found a prevalence of PPD > 4 mm in 22% of athletes vs. 19% within the population control group. Generally, when information on periodontitis was provided, prevalence ranged from low (0–5%) [15,19,26] to more frequent (15–41%) [10,17,21,27,29,30].

### 2.4. Impact of Dental Status on Performance and Quality of Life

Five studies measured the impact of dental status on performance and quality of life, using self-reported questionnaires (Table 1). Samples were taken from athletes attending dental clinics [10,16,27] or an assessment of whole teams [19,21]. Athletes reported a negative impact of their oral health on performance (6–18%) and quality of life (QoL; 20–28%). The oral cavity provides a habitat for pathogens and a window for systemic infections. Local oral inflammation can cause a systemic spread and thus affect physical performance [35,36,37,38].

Poor oral health in athletes including caries, erosion, and periodontal diseases is not a new finding. This is in contrast with the common perception that athletes are healthy due to physical exercise. The positive effects of physical training on systemic inflammation [39,40], the prevalence of periodontitis [41], and decreased odds of periodontal disease were reported [42,43]. Weight loss in combination with 3 months of training in obese subjects improved periodontal disease [44,45]. A positive relationship was found between periodontal/endodontic disease and reduced physical fitness [46]. In turn, high physical fitness levels corresponded to a lower risk for periodontitis in 40,000 males [47], and low cardio-circulatory fitness to moderate and severe periodontitis [12,48]. In a recent study, long-term physical training improved clinical signs of periodontitis in type 2 diabetic patients [49]. This was explained by a training-induced increase in gingival microperfusion and local oxygen supply [49]. 

## 3. Reasons for Poor Oral Health in Athletes

So, how can this apparent contradiction between the favorable effects of physical training on periodontitis and the rather increased occurrence in competitive sports be explained? Three major reasons for poor gingival and periodontal health, but also for poor dental health in competition sports, are presented in the following paragraph: oxidative stress, sports diet, and oral hygiene.

### 3.1. Oxidative Stress and Oral Health

Several studies measured increased levels of oxidative stress markers in blood, saliva, and crevicular fluid in cases of periodontitis, supporting the association between oxidative stress and periodontal inflammation [50,51,52,53]. The advancement of periodontitis may be favored by oxidative stress. Reactive oxygen species (ROS) are components of normal cellular metabolism and play an important role in signal transduction and immune defense. An excess of ROS or chronic oxidative stress, however, contribute to increased oxygen load and decreased antioxidant mechanism ability to neutralize ROS, which leads to cell and tissue destruction [50,54,55,56]. ROS damage biomolecules and cell membranes [57], and impair antioxidant factors [58,59], which correlates with the development and progression of periodontitis [60]. ROS lead to an enhanced expression of pro-inflammatory cytokines and extracellular connective tissue destruction, causing dental attachment loss, bone resorption, and finally periodontal disease [57,61,62,63]. The relationship between exercise and oxidative stress depends on the type of exercise, intensity, and duration. Regular moderate training seems to foster oral health and protect against oxidative stress [49,64]. In contrast to aerobic exercise, high-intensity training can increase oxidative stress [64]. Oxidative stress may impair the immune system in high-performance athletes with high cortisol levels due to stress, demand, and overload [11,12], which can have a negative impact on oral and dental health. High intakes of carbohydrates can promote oxidative stress and inflammatory responses [65,66]. A recent study showed no significant differences in salivary oxidative stress biomarker values in patients with implants and controlled periodontitis compared to healthy controls [56]. However, the analysis of biomarkers for oxidative stress in saliva is not sufficient at the current state of research to reliably detect tissue damage.

### 3.2. Sports Diet and Oral Health

Sports diet affects the type, frequency, and quantity of carbohydrate intake, but also the sport-specific composition of protein and fat. The respective proportions of these components can have different effects on dental and oral health.

Carbohydrate substitution is particularly important for endurance sports, but also for sports that require a lot of energy. Therefore, a major reason for poor oral health is frequent high sugar/carbohydrate nutrition [8,20]. Athletes have a high energy requirement to maintain their weight and body composition [67]. With energy deficiency, fat and lean tissue will be used for metabolism. The loss of lean tissue is detrimental and leads to a loss of endurance and power as well as to an impairment of the immune, endocrine, and musculoskeletal systems [68]. The energy expenditure depends on training duration, frequency, and intensity, as well as on gender, genetic susceptibility, age, height, and fat-free mass. The considerations of sports diets primarily focus on an optimal intake of carbohydrates, fats, proteins, and minerals [4], but also have a secondary effect on oral mucosa and dental hard tissues [69]. The quality and type of nutrition, physicochemical properties of the saliva, and eating patterns have a pivotal influence on the microbiota’s composition and properties [70,71].

### 3.3. Carbohydrates in Sports and Caries

Prolonged exercise induces the depletion of muscle glycogen stores. Frequently, sports diets focus on increasing glycogen stores as well as on additional carbohydrate (CHO) supply during physical strain. A recent study categorized 28% of elite and professional athletes as high sugar consumers, 59% reported the use of energy bars, and 70% noted the use of energy gels [27]. 

The composition of oral microbiota is closely linked to the salivary pH level which depends on the availability of food debris and fermentable carbohydrates, fostering the multiplication of aciduric species. In common, the oral and dental health defense status is related to salivary flow rate and pH level, buffering capacity, plaque biofilm, cariogenic bacteria, and host immune defense/immunoglobulin A (IgA) levels [72]. 

Carbohydrates are classified into sugars and starches. These fermentable carbohydrates represent the most important substrate for bacterial metabolism. The adhesiveness, solubility, and texture affect the sugar clearance by salivary flow. Prolonged oral retention of foods lead to an extended period of acid production. The more frequently food and drinks are consumed and the shorter the intervals, the greater the risk of dental damage. Caries is caused by the interaction between the host, bacteria, carbohydrate availability, and time [73].

A high frequency of sugar consumption creates an imbalance in the oral microbiota [74]. A large number of studies have characterized the microbiome with respect to dental caries and periodontal disease [75]. The ecosystem in the biofilm is considered a decisive factor for tooth decay. Thus, caries and periodontal disease arise from plaque biofilm imbalance [76,77]. The organic acids produced by cariogenic bacteria dissolving the dental hard tissue may lead to cavitation if this process is prolonged and frequent [78]. 

The connection between dental caries and carbohydrates has already been proven [79,80,81]. A correlation was found between sugar consumption and higher caries experience [82], especially in cases of sugar consumption between meals [83]. The frequency of sugar consumption [84,85] as well as the amount influences the development of dental caries [86,87], whereby the latter appears to be more cariogenic according to some studies [88,89,90]. The type of sugar and food is crucial. Sucrose is more cariogenic than other sugars (e.g., fructose, maltose, lactose, and glucose) [79,87,91]. A pH of 5.5 is considered the “critical pH” for enamel loss [92], and starch-containing foods can reduce the pH level even more [93]. 

The local dietary effects on the teeth are dependent on the frequency and amount of carbohydrate/sugar intake and are affected by the intra-oral environment such as overall dietary regimen, biofilm and saliva composition, saliva flow, tooth morphology, oral hygiene, and fluoride [94]. The likelihood that an athlete consuming a lot of carbohydrates will develop caries, gingivitis, and/or periodontitis depends on preventive factors and behaviors. The negative effects of the dental plaque may be enhanced in athletes due to mouth breathing and dehydration, pro-inflammatory effects of frequent carbohydrate consumption, and a weakened immune system by intensive exercise load and stress [6,95]. The development of caries is influenced by the balance between caries-promoting and preventing factors. The primary dietary nutrients with an increased caries risk are sugar-containing drinks and energy drinks, sticky foods, sugary–starchy snacks, and simple sugars, with frequent and prolonged eating habits. Dietary nutrients with decreased risk are sugar-free products, fresh fruits and vegetables, and whole-grains, with a time frequency of food and beverage intake at least 2 h apart [96].

### 3.4. Carbohydrates and Periodontal Disease

Oral diseases cause increased levels of inflammatory cytokines, which have a profound impact on the development of muscle fatigue and oxidative stress [97,98,99,100]. Muscle fatigue increases the risk of muscle cramps and proprioceptive dysfunction [101,102], resulting in an increased risk of sports injuries [103]. Muscle injury was associated with plaque accumulation and PPD in professional male soccer players [15]. A higher prevalence of self-reported muscular and articular injuries in professional football players with periodontitis was reported [30].

Periodontitis is a multifactorial inflammatory disease of the periodontal tissues in which oral bacterial flora, lifestyles (e.g., oral hygiene, diet, and malocclusion), and genetic factors can affect individual susceptibility [104,105]. The specific bacteria associated with periodontitis are Porphyromonas gingivalis, Tannerella forsythia, Prevotella intermedia, Fusobacterium nucleatum, Treponema denticola, and Actinobacillus actinomycetem comitans [106,107].

However, nutrition is also indirectly responsible for periodontal health [108,109,110,111,112]. High carbohydrate consumption has pro-inflammatory effects and thus increases the risk of periodontal inflammation [113,114]. A Stone Age diet for 4 weeks without dental cleaning showed better gingival conditions, despite the increase in dental plaque [113]. However, plaque biofilm is considered the most important factor, and thus, oral hygiene is pivotal. High sucrose consumption is associated with increased plaque volume, which fosters gingivitis and gingival bleeding [115,116,117,118]. This was even seen in people with an apparently excellent oral hygiene regimen. 

The underlying mechanisms of increased periodontal inflammation may be found in elevated levels of glucocorticoids/stress hormones in athletes combined with impaired saliva immune defense. Increased levels of glucocorticoids result in macrophage dysfunction and lower cytokine levels, which affects periodontal health [119]. In addition, there is a reduced flow of saliva, which decreases salivary immunoglobulins A and antimicrobial proteins (α-amylase, lysozyme, and lactoferrin), resulting in susceptibility to oral inflammation [120]. 

### 3.5. Proteins in Sports and Caries

Recommendations for daily protein intake among athletes are between 1.2 and 2.0 g/kg/day [121,122]; furthermore, the sport intensity, individual regulations, and requirements must also be considered. A higher consumption during intensive training may result in additional benefits [122,123] due to upregulated muscle protein synthesis with an increased sensitivity to protein ingestion during 24 h post-exercise [121,124]. 

High-quality protein foods (meats, eggs, cheese, fish, and vegetables) are associated with a decreased risk for dental caries [97]. Milk is rich in protein, which provides essential amino acids and organic nitrogen for athletes. Milk contains two major proteins (casein at 80% and whey at 20%), as well as enzymes, vitamin-binding proteins, and growth factors [125].

Milk-derived protein activity inhibits bacterial enamel binding, supports buffering, and enhances enamel remineralization [126]. These antimicrobial effects are also used in commercial products such as toothpaste, gels, mouth rinses [127,128], and chewing gum. Dairy products contain calcium, phosphate, and lipids, which have caries-protective effects [129,130,131]. Cheese harbors casein phosphor-peptides to stabilize calcium and phosphate to amorphous calcium–phosphate, a special textural effect in binding calcium and phosphate in solution, as well as in dental plaque and enamel [132,133,134,135]. Chewing hard cheese stimulates the salivary flow and remineralization of the teeth by increasing calcium and phosphate in the dental plaque [134,136]. The enamel remineralization effect could also be achieved with soft cheese [137], and even processed cheese was claimed to be anticariogenic [138]. Cheese contains the amino acid tryptophan, a component of the euphoric mood substance serotonin, which plays an important role in inducing and maintaining sleep. Foods containing tryptophan, such as soy flour, dairy products such as cheese (especially hard cheese), meat, fish, and nuts, can support healthy sleep. In addition, cereals, potatoes, or rice promote the production of serotonin in the body as well as carbohydrate-rich foods/sugars. But the latter have cariogenic and pro-inflammatory effects as described above. Some studies suggest that certain cheese ingredients, such as peptides produced during fermentation, suppress inflammation in the brain and prevent cognitive decline. Inflammation in the brain is regulated by microglia. Tryptophan-related dipeptides in fermented dairy products may suppress microglia activation and thereby prevent cognitive decline, but the underlying mechanisms remain to be clarified and further research is warranted [139].

As a result of strength and endurance sports, there is a higher need for protein, which the athlete covers through specific protein intake in accordance with dietary recommendations. This means that athletes generally consume more protein, which has an overall anticariogenic effect, but studies have shown that athletes tend to have poor dental health. The incidence of tooth decay in athletes is higher than in the average population despite an obviously higher protein intake. However, this contradiction may also indicate that the timing of protein intake in combination with cariogenic foods must be chosen well. For example, it may be beneficial to consume protein products at the end of a meal or to drink more milk instead of sugary drinks. For example, buttermilk is the ideal sports drink due to its composition. Cheese and milk have a protective potential against teeth demineralization [137,140].

### 3.6. Proteins in Sports and Periodontal Disease

Proteins also positively affect periodontal health. Studies showed an inverse association between protein intake and the prevalence of periodontitis [141,142,143]. The gingival tissue has one of the highest turnover rates in the body, and proteins are crucial for structural maintenance. Severe protein malnutrition causes tooth loss and periodontal lesions [144]. An inadequate intake of proteins negatively effects the immune system, wound-healing, and antibacterial properties of the saliva [96]. As previously mentioned, the simultaneous or more frequent consumption of sugary products could also negate this positive effect in the context of athletes’ sports diets. 

### 3.7. Fats in Sports and Caries

The proportion of fat in the sports diet is around 20–35%. Fats are components of cell membranes and nerves [145]. A fat restriction to <20% of total energy means a low intake of fat-soluble vitamins and essential fatty acids (omega-3) [121,145,146]. 

Body composition and weight can affect athletic performance and are criteria for participation in sports. A high body mass index (BMI) is associated with a high risk for periodontitis [147], which can play a role in heavy weight sports. Optimal body-fat levels depend upon heredity, age, gender, and practiced type of sport [5].

A caries-protective effect may be attributed to essential fatty oils (Eos) from aromatic plants [148,149]. Monoterpenes are the major compounds found in Eos [148] and show antibacterial effects against caries-related microorganisms, particularly Streptococcus mutans [149,150,151]. Oil pulling has been used to treat caries, oral malodor, and gingival bleeding [152,153,154]. The anticariogenic effect may be attributed to a reduction in Streptococcus mutans bacteria and an inhibition of bacterial adhesion [155]. 

### 3.8. Fats in Sports and Periodontal Disease

Diet can alter the cell membrane and blood lipid profiles [156,157], and thus the susceptibility to oxidative damage [158]. The defense against bacterial attacks was also modified by membrane lipid profile [159]. Based on these observations, changes in dietary habits that affect lipid profiles may be interesting to prevent and improve periodontal diseases [160,161]. Polyunsaturated fatty acids (omega-3) promote periodontal health through their antioxidant and anti-inflammatory effects. In contrast, saturated fat-rich diets increase oxidative and inflammatory stress [161]. So, the type of fat is crucial for general systemic and periodontal health. A low-carbohydrate and high-fat diet modified the oral microbiota among endurance walkers, resulting in decreased relative abundances of *Haemophilus*, *Neisseria*, and *Prevotella* and increased *Streptococcus* spp. [162]. In terms of the promotion of anti-inflammatory mediators, strict attention should be paid to a sufficient and balanced ratio of omega-6 to omega-3 fatty acids (≤5:1). A lack of omega-3 fatty acids depresses the anti-inflammatory and immune response of periodontal tissue.

### 3.9. Sports Drinks, Saliva, and Dental Erosion

Athletes require also additional fluid to cover sweat losses. Gallagher et al. [27] found that 86% of athletes consumed sports drinks during training/competition. Needleman et al. [10] reported a consumption of sports drinks of at least three times per week in 64% of athletes. In triathletes, Bryant et al. [25] reported that sports drinks were consumed in 84% of athletes while training. Sports drinks contain carbohydrates to maintain blood glucose levels and fluid balance under endurance load [5]. Sweet stimuli activate central feeding circuits and reward reflexes, which further stimulate the appetite for sweets [163]. Studies reported that flavored or sweetened beverages can increase the voluntary intake of sugar during and after exercise [164,165]. Sports beverages include energy drinks (>10% CHO), isotonic sports drinks (4–8% CHO), and hypotonic drinks (2% CHO or less) [166].

An overall significant effect of CHO mouth rinse on performance has been reported. The glucose mouth rinse (without ingestion) during endurance exercise stimulates oral taste receptors, which are associated with central nervous motivation pathways for performance enhancement [40,167] by modulating the central governor theory [168]. A systematic review documented that mouthwash with a glucose solution (6–10%) every 5–10 min for 5–10 s may increase the performance by 2–3% [38] in high-intensity loads/cycle ergometry sessions up to 1 h [169]. In contrast, other studies found no effects of glucose mouth rinse on resistance training (bench press) after an overnight fast [170] and on cycle ergometry performance post-prandial (breakfast) [171]. However, frequent tooth contact and repeated rinsing with sugary sports drinks have negative consequences for tooth and gum health, which are specified in the following section. 

Normal saliva is highly saturated with calcium and phosphate, favoring remineralization [79]. Other components are many proteins, electrolytes, and substances from blood and alveolar fluid [172,173]. The determinants of a physiological salivary pH are composition and flow. The normal flow for unstimulated saliva is above 0.1 mL/min; values below indicate hypofunction and values of 0.2 mL/min or more apply to stimulated salivary flow [172].

Factors which decrease the salivary pH and promote dental damage are poor oral hygiene, acidic and sugary drinks, acidic foods (citrus fruits), chewable vitamin-C tablets, and reduced salivary flow [173,174].

Sports drinks contain minerals, electrolytes, acids (citric, phosphoric, ascorbic, malic, tartaric, and carbonic acids), and carbohydrates. Their consumption results in lower salivary pH values, and enamel dissolution starts at 5.5 [92]. The dentine or root surfaces have a lower mineral content and therefore a higher critical pH of approximately 6.2 for dissolution [144]. 

Commercial sports drinks have a pH value between 3.2 and 3 [175], but the contact time with dental enamel is decisive for irreversible damage. Prolonged consumption favors dental erosion [176,177,178]. Under normal circumstances, acidic fluids are eliminated within 10 min [179], but with low salivation, this can take up to 30 min [180]. There is evidence that exercise decreases salivary flow rates and causes dehydration and thus an increased viscosity of the saliva, resulting in decreased buffering and antibacterial properties. After a two-hour cycle ergometry session, salivary flow was decreased by 39% [181] and saliva IgA secretion by 19.5% [175]. These exercise-induced factors result in dental erosion, decreased host immune response, and increased susceptibility to oral cavity pathologies [26,182,183,184].

It has been shown that the frequency of acidic drink consumption is more crucial for dental erosion than the total amount [185]. Athletes with a frequent consumption, decreased salivary flow, prolonged drinking patterns, or mouth breathing are especially at risk of dental erosion [186]. Additionally, the exercise intensity itself further impairs the defensive properties of the saliva up to 2 h post-exercise. Another key aspect in addition to the direct effects of acidic drinks described above is the sugar content of sports beverages and the indirect formation of acids by oral plaque bacteria due to sugar fermentation. Thus, organic acid produced by oral plaque microorganisms results in the demineralization of teeth and caries [187]. Sugary sports and energy drinks can have a lower pH than 3. Therefore, athletes should avoid sugary beverages and supplements outside of training, competition, or recovery. After sports drink intake, a subsequent mouthwash with plain water is useful to increase salivary pH value. In cases of high caries and erosion risk, dentists generally recommend using an anticaries mouth rinse containing fluoride to reinforce the structure of the enamel. Athletes who frequently use sports drinks can implement this mouthwash as part of their dental care at home and during training. Chewing gums can stimulate the salivary flow and consequently improve the buffering capacity due to higher bicarbonate levels. However, this recommendation cannot apply without restriction, as chewing can also increase the risk of temporomandibular joint problems or muscle pain. Benefits and risks must be carefully considered.

### 3.10. Antioxidants, Dental Caries, and Periodontal Disease

Polyphenol antioxidants (e.g., flavonoids, phenolic acids, and carotenoids) contained in fruits (e.g., dark berries) and vegetables (e.g., dark leafy greens) have been considered potentially anticariogenic [188,189] as they reduce caries pathogenic bacterial growth and biofilm formation [190]. But these studies mostly used single-species biofilms, and no clinical evidence has demonstrated a real anticariogenic benefit [191].

Recent studies showed that reduced levels of antioxidant micronutrients affect periodontal health in cases of gastrointestinal disorders, poor diet, or lifestyle [140]. A sport-related diet should contain several antioxidants, such as vitamin A, C, and E and glutathione, which promote periodontal health [192,193]. Vitamin A is a fat-soluble vitamin and has been used to supplement periodontal treatment [192,194], showing slight improvements. It stimulates salivary flow and thereby helps to stabilize salivary pH levels [195]. Dietary sources of vitamin A include eggs, carrots, liver, sweet potato, broccoli, and leafy vegetables. In veganism, there is a risk of vitamin B12 deficiency. A prospective cohort study reported low serum B12 levels in cases of worse periodontal status [196]. Vitamin C is required for collagen synthesis [197], and it also enhances iron absorption [198] and promotes tissue healing [199]. Compared to conventional toothpastes, it was found that a toothpaste containing vitamin C can improve gingivitis and anti-ROS effects [200]. A long-lasting vitamin C deficiency leads to scurvy. The risk of periodontitis increases by 20% with low vitamin C intake [201]. Vitamin D deficiency may decrease periodontal attachment [197] and jaw-bone density, but further studies are necessary to clarify the association between serum vitamin D values and periodontal health [202]. The impact of vitamin E on periodontal health needs also further research. Green tea or green tea extract with caffeine have even more [203] antioxidant potential to scavenge ROS [204,205]. The health benefits of consuming phenols derive from synergistic effects between bioactive compounds and other nutrients in fruits or vegetables. Cooking may alter the antioxidative properties [206]. 

ROS and free radicals are predominant in periodontitis [207]. Antioxidants may inhibit ROS-mediated periodontal inflammation [208,209]. A restricted dietary intake of antioxidants compared to a conventional diet over 2 weeks increased systemic oxidative stress markers in athletes by 38% after submaximal exercise, 45% after exhaustive exercise, and 31% after 1 h recovery. Therefore, a balanced diet rich in natural antioxidants and polyphenols is recommended [210].

### 3.11. Probiotics, Prebiotics, Oral Health, and Sport

Probiotics are used as natural components to prevent gastrointestinal problems. Probiotics are preparations containing microorganisms, for example lactic acid bacteria and yeasts. Prebiotics (such as inulin and oligofructose) are dietary fibers that promote the growth and activity of bacteria in the large intestine [211,212]. Dietary fibers stimulate primarily the proliferation of bifidobacteria and lactobacilli. Probiotics are natural components of fermented foods as yogurt, kimchee, and sauerkraut. The intestinal microbiota has an impact on host metabolism, physiology, nutrition, and immune function [213]. The fermentation of dietary fiber ingested with food is carried out by intestinal bacteria (e.g., anaerobic degradation of short-chain fatty acids such as acetate, propionate, and butyrate). The short-chain fatty acids stabilize intestinal bacteria, stabilize the function of the intestinal barrier, and also have systemic metabolic effects. Further beneficial effects of these short-chain fatty acids are the regulation of appetite and metabolism as well as cancer prevention. The increase in short-chain fatty acids causes the intestinal pH value to drop slightly, creating an ideal climate for the proliferation of bifidobacteria. A healthy intestinal microbiota is characterized by a high diversity of bacteria. Intestinal bacteria are also involved in the production of vitamins (e.g., vitamin K, B-complex, and fatty acids), activation of the immune system, and reduction in systemic inflammations. [214]. Studies that focus specifically on the relationship between probiotics and sport reported possible benefits. Probiotics impaired the occurrence of cold-like symptoms after intensive exercise load [213]. In reducing the risk of developing infections or the severity of related symptoms in athletes, the majority of studies reported beneficial effects [215]. Other studies revealed a symbiotic association between oral bacteria and an entero-salivary nitrate–nitrite–nitric oxide pathway, which supports nitric oxide (NO) homeostasis [216,217]. NO has physiological respective biochemical effects on vasodilatation, neurotransmission [218], immune defense [219], oxidative energy regeneration [220], and the muscle contraction process [221]. Inorganic nitrate is a natural micronutrient (e.g., green leafy vegetables), but human cells can only activate biologically inert nitrate to a very limited extent. The bioavailability of NO is increased by symbiotic oral bacteria. They reduce ingested nitrate to bioactive nitrite [222], which can be reduced to NO in the circulation or in regions of low oxygen availability [223]. This benefit may be negatively affected by an imbalanced oral microbiota [223]. A diet with a high nitrate content, which focuses on the consumption of vegetables, leads to an increase in nitrate and nitrite concentrations in the bloodstream, positively affecting blood vessels. It is therefore also conceivable that nitrate supplementation leads to a faster acceleration to maximum speed in high-intensity sports [223].

### 3.12. Probiotics, Dental Caries, and Periodontal Disease

Probiotics can help to treat dental diseases originating from infections/microbiota imbalances [224]. Probiotics may enhance the proliferation of nitrate-reducing bacteria and NO production [225]. Probiotics can adhere to and colonize various surfaces of the oral cavity [226] and are used as an anticaries and anti-periodontitis agent [144]. The effects of probiotics on periodontitis or its development are ambiguous. Some studies have shown positive effects of pharmaceutical probiotics on pocket depth in periodontitis [227], bleeding on probing, and inflammation [228,229]. Probiotic dairy products (e.g., milk, yoghurt, kefir, curd, and cheese) or pharmaceutical formulas can improve oral health by modifying the microbiota (decrease in Streptococcus mutans levels) [230]. Several studies found that probiotics positively influence caries and periodontitis development [229,231,232,233,234,235]. Their continuous supply also seems to be decisive for the effect. Discontinuing consumption will diminish these positive effects after 2–4 weeks [234]. Further research is necessary to clarify how exercise training influences the oral microbiome. The latest research has unveiled a positive role of probiotics in the prevention of caries, halitosis, and periodontitis. Therefore, the supplementary use of probiotics apparently plays a relevant role in athletes’ dental care.

### 3.13. Oral Hygiene Behavior

The effects of carbohydrates and acidic/sugary drinks on oral health are enhanced by exercise training. Dehydration and local mouth drying during exercise reduce salivary flow and therefore impair remineralization and antimicrobial activity, in addition to exercise-induced immune suppression. Nutrition, including sports diet, beverages, and supplements, is a major determinant for oral health. High carbohydrate intake is one of the pivotal causes of tooth decay and its pro-inflammatory effects contribute to the development of periodontal inflammation. Acidic sports drinks can cause dental erosion, as in eating disorders. Teeth brushing immediately after acidic drink consumption increases tooth surface loss [236]. Rinsing with plain water or milk after acidic sports drink intake reduces contact time and dental damage by neutralizing oral pH levels more quickly [6,8]. A simple risk mitigation strategy is to use a “two-bottle strategy” (sport supplement, followed by plain water) [6]. A further recommendation is the use of a dentifrice containing tin and fluoride, which showed a higher effectiveness against dental erosion than a dentifrice with only tin or fluoride [237,238]. Where carbohydrate or sugars are consumed regularly, fluoride toothpastes or high-concentrated fluoride toothpastes (2800 ppm or more) should be used twice per day to reduce dental erosion. To maximize the fluoride uptake, no intense spit or rinse after tooth brushing is recommended [6]. Fluoride ions, incorporated into the enamel surface, protect against dental erosion. Furthermore, together with the calcium-containing saliva, a calcium–fluoride precipitate on the tooth surface can be established which protects against dental caries [239]. Additionally, a sodium fluoride mouth rinse (0.05%) at a different time of the day is recommended, as well as local fluoride application (gel or varnish) [6]. Chlorhexidine mouth rinse was reported to impair the nitrate to nitrite conversion for ergogenic supplementation [240,241], the plasma nitrite level increase, and blood pressure decrease [242,243]. Therefore, oral microbiota and dental hygiene behavior have effects on oral health and sports performance. Intense oral hygiene including interdental cleaning is crucial to reduce the dental plaque biofilm. The use of self-disclosing solutions is a supporting tool for plaque visualization. The athlete should take responsibility for daily oral health self-care.

## 4. Limitations

The data search concerning athletes’ oral health was limited to elite or professional athletes and only 17 studies were found. Outcome measures of oral health concerning the prevalence, incidence, and/or severity of dental caries, dental erosion, gingivitis, and periodontal disease were included. In principle, data from quantitative clinical and/or radiological examinations were preferable. However, only a few studies were available and mostly with a lack of representative sampling.

Disease incidence was generally not clearly differentiated by the socioeconomic status; however, athletes from developing and developed countries were affected. Only six studies related the data to a comparison group of amateur or non-athletes, and three studies to a population survey. Moreover, there was a lack of information on the risk of dental health in relation to certain types of sport or weekly training volume. Although several studies had been cited on the issue of oral health in athletes, a precise assessment remains to be made. The studies showed only inhomogeneous results, which is why they were described here in a narrative form. For the future, prospective longitudinal studies with elite athletes of different disciplines and age groups with respect to a high age-dependence of some oral parameters will be important. Comparison groups were largely missing, so a comparison of larger samples to age-matched regional control groups will help to determine risk levels and determinants of oral health. Questionnaires concerning nutrition, stress level, and oral hygiene should be additionally collected to explore the reasons for poor oral health. Furthermore, the relationship between nutrition and oral health in sport was presented in the current comprehensive overview of various aspects and from many perspectives. Based on this, specific indications for future research and perspectives in the context of oral health and nutrition in competitive sports are presented. But this is only one side of the coin. On the other side, the topic of degenerative/chronic inflammatory diseases in the context of physical activity and nutrition would also be interesting.

## 5. Conclusions

Three main aspects influence oro-dental health in sport: reduced dental care, sport-specific diet, and nutritional modalities, as well as sport-related pathophysiological components such as systemic inflammation and reduction in salivary flow. One core problem is that the nutritional requirements of competitive sports are in many respects in conflict with the requirements of oral health. The structural basis of a sports-specific prevention program is knowledge and behavior. Knowledge concerns, e.g., all possible side effects of nutrition components and consumption modalities on oro-dental and systemic health, but also the underlying pathophysiological mechanisms and their effects on sports performance. Behavioral components are all managing strategies like avoidance of prolonged sugar contact, sticky adhesive carbohydrates, and supplements not benefiting performance. Further aspects are matching sports drinks to purpose, rinsing with plain water or milk after carbohydrate or acidic exposure, and increasing salivary flow and pH level. 

Saliva analysis could be applied to choose the best diet and training regimen for the athlete. Salivary oxidative stress biomarkers are currently being researched to detect oral tissue damage. Their analysis is a promising non-invasive and simple method and may replace frequent oral PPD measurements in the future. Overall, good oral hygiene practices, oral health promotion, routine periodic assessment every six months, educational interventions, and personalized dental care instructions are necessary for the athletes to increase their oral health and to reduce the risk from the sports diet of oral and dental diseases.

## Figures and Tables

**Table 1:** Summary of studies reporting oral health status in elite athletes and its impact on performance, training, and quality of life.

Ref.	Study Design	Athletes Cohort	Sample Size [Total (F/M)]	Age [Mean (Range, SD)]	Control Cohort	Sample Size [Total (F/M)]	Age [Mean (Range)]	Measurement	Comparison	Impact on Oral Health (SD)	Negative Reported Impact on Performance/P, Training/T, and Quality of Life/QoL
[23]	C	Soccer players from Brazil; competitive sport.	18 F	(13–19)	n.d.			Oral examin.	n.d.	DMFT: 7.15	n.d.
[24]	RC	Commonwealth Games 2010; athletes from India (74), Kenya (35), Nigeria (33), America, Africa, Asia, and Australia; competitive sports.	342 F/M n.d.	n.d. >18	n.d.			Dental treatment	n.d.	Filling/endodontic treatment: 22%	n.d.
[25]	C	Triathletes from New Zealand; competitive sport.	31 M	n.f.	n.d.			Questionnaires; 10 oral examins	n.d.	High caries risk DMFT: 0–4 in 6 athletes, and 9 in 2 athletes	n.d.
[15]	Cvs.CC	Soccer players from Barcelona; competitive sport.	30 M	21 (1.6)	Dental and medical students	n.m.	similar	Oral examin.	Active caries: 1 DMFT: 5 DMFT: 3.4	Active caries: 2.2 DMFT: 5.7 GI: 1.1 PD: 0%	n.d.
[10]	RC	Olympic Games London; athletes from Africa, America, Europe, and Asia; competitive sports.	278 (119/159)	25.7 (16–47)	n.d.			Attendance at dental clinic	n.d.	Caries: 55% ER: 45% GI: 76% PD: 15% (PPD > 3)	T/P: 18% QoL: 28%
[20]	Cvs.CC	Triathletes from Germany; competitive sport.	35 (11/24)	36.8 (21–48)	Non-exercising people	27 (3/24)	36.1 (23–52)	Oral examin.	DMFT n.d. BEWE: 7.3	DMFT: 9.4 BEWE: 9.6 *	n.d.
[21]	C	Professional soccer players from Thailand; competitive sport.	25 M	27.5 (4.7)	n.d.			Oral examin.	n.d.	DMFT: 10.1 Caries: 84% PD: 30%	P: 18% QoL: 28%
[26]	Cvs.CC	Italian swimmers; competitive sport.	54 (26/28)	12.5 (3.3)	Non-competitive swimmers	69 (32/37)	9.9 (3)	Oral examin.	n.s.	DMFT: 0.08 GI: 0.05 ER: 1%	n.d.
[19]	Cvs.CC	Professional British football players; competitive sport.	187 M	24 (18–39)	Population data			Oral examin.	Caries: 30% Other: n.d.	DMFT: 4.6 Caries: 38% ER: 53% GI: 77% PD: 5% (PPD > 3)	P: 7% QoL: 20%
[18]	Cvs.CC	Professional rugby players from France; competitive sport.	24 M	27.3 (4.7)	Non-exercising	22 M	26.6 (3.9)	Oral examin.	DMFT: 2.1 GI: 13.6%	DMFT: 5.5 * GI: 58% *	n.d.
[27]	Cvs.CC	Professional and Olympic athletes; competitive sports.	352 (116/236)	25 (18–39)	Population data			Oral examin.	Caries: 36% PD: 19%	Caries: 49% ER: 42% GI: 77% PD: 22% (PPD > 3)	P: 6% T: 4%
[16]	Cvs.CC	Olympic Dutch athletes; competitive sports.	116 (76/40)	25.8	Population data			Oral examin.	DMFT: 3.1 ER: 22% Other n.d.	DMFT: 3 Caries: 20% ER: 59% * BEWE: 2 GI: 64% PD: 1%	P: 10% QoL: 27%
[28]	Cvs.CC	Athletes from Middle East; competitive sports.	1079 F/M n.d.	21.7	Non-athletes F/M n.d.	116 F/M n.d.	22.4	X-ray interpretation	Caries: 85.5%	Caries: 89%	n.d.
[29]	C	Pan American Games 2019; athletes mostly from Middle and South America; competitive sports.	76 F/M n.d.	>18 n.d.	n.d.			Attendance at dental clinic		Caries: 29% PD: 34% (PPD > 3)	n.d.
[30]	C	Professional football players from Portugal; competitive sport.	22 M	27.7	n.d.			Oral examin.		PD: 41% (PD > 3)	n.d.
[17]	RCvs.CC	German elite athletes; competitive sports.	88 (45/43)	20.6 (3.5)	Sports students	57 (29/28)	22.2 (2.1)	Oral examin.	DMFT: n.s. Caries: 19% BEWE: 3.5 PD: 12%	DMFT: 2.7 Caries: 34% * BEWE: 3.5 PD: 40% *	n.d.
[22]	C	Special Olympic Games; Italian athletes with Down syndrome; competitive sports.	171 (63/108)	26.2 (16–54)	Athletes with intellectual disabilities	170 (78/92)	28.5	Oral examin.	DMF: 10.5 Caries: 52%	DMF: 9.7 Caries: 38.6% GI: 60%	n.d.

Conclusions: two different categories were found for studies among professional/elite athletes: 1. Epidemiological surveys were used to determine the prevalence of a specific dental condition (82%). Studies were characterized by oral examination or by screening athletes or whole teams for a specific condition with (an) independent examiner(s). 2. Dental problems or treatment audit to determine the prevalence or incidence was determined through attendance at a dental clinic or by analyzing retrospective data from treatment records (18%). In general, the methodological quality and sampling was low (n:18–54). Six studies related the data to a comparison group of amateur- or non-athletes, and three studies to a population survey. The lack of consistency in the outcome measures between the studies made the comparison of results difficult. Most of the studies recorded caries as a proportion; it ranged from 15% to 89% of all athletes. BEWE: erosive tooth wear; C: cohort; CC: case–control; DMF(T): decayed, missing, and filled teeth/index; ER: erosion; F: females; GI: gingivitis; n.d.: not determined; n.m.: not mentioned; n.s.: not significant; n.f.: not found; Oral examin.: oral examination; P: performance; PD: periodontal disease; PPD: periodontal pocket depth; QoL: quality of life; RC: retrospective cohort; Ref: references; *: significant; SD: standard deviation; T: training.

## Abbreviations

BMI	Body mass index.
BEWE	Erosive tooth wear.
BOP	Bleeding on probing.
BPE	Basic periodontal examination.
BPI	Periodontal bleeding index.
CHO	Carbohydrates.
DMF(T)	Decayed, missing, and filled teeth/index.
ER	Erosion.
Eos	Essential fatty oils.
ETW	Erosive tooth wear.
F	Females.
GI	Gingivitis.
HDL	High-density lipoprotein;
IgA	Immunoglobulin A.
LDL	Low-density lipoprotein.
M	Males.
NO	Nitric oxide.
P	Performance.
PD	Periodontal disease.
PPD	Periodontal pocket depth.
PSI	Periodontal screening index.
QoL	Quality of life.
Ref	Reference.
ROS	Reactive oxygen species.
SD	Standard deviation.
T	Training.
